# Hematology, Biochemistry, and Blood Gas Reference Intervals for Captive Anesthetized Long-Tailed Goral (*Naemorhedus caudatus*)

**DOI:** 10.3390/ani15091216

**Published:** 2025-04-25

**Authors:** Ockju Im, Suk-Jin Lee, Hong-Cheol Kim, Jeong-Jin Yang, Jang-Ik Son, Doo-Ha Yang, Dong-Hyuk Jeong

**Affiliations:** 1 Laboratory of Wildlife and Conservation Medicine, Colleage of Veterinary Medicine, Chungbuk National University, Cheongju 28644, Republic of Korea; uroconure@gmail.com; 2 Korea National Park Institute for Wildlife Conservation, Gurye 57616, Republic of Korea; mdungus5@knps.or.kr (S.-J.L.);; 3 Wildlife Center of Chungbuk, Cheongju 28116, Republic of Korea

**Keywords:** long-tailed goral, *Naemorhedus caudatus*, hematology, biochemistry, blood gas analysis, reference interval

## Abstract

The long-tailed goral (*Naemorhedus caudatus*) is classified as an endangered species globally, and national projects for conservation are underway in South Korea. However, blood test references would enable comprehensive health assessments in this species. This study provides reference intervals for hematology, biochemistry, and blood gas analysis in long-tailed goral for health assessments and to aid conservation strategies. Blood test data from 75 gorals managed between 2016 and 2020 were analyzed to establish reference intervals and to examine the influence of age and sex on hematological and biochemical parameters, as well as differences between arterial and venous blood in blood gas analysis. Significant age-related differences (*p* < 0.01) were observed in MCH, lymphocyte count, platelet count, total cholesterol, HDL cholesterol, ALT, amylase, creatinine, calcium, and inorganic phosphorus. Sex-related differences were found in lymphocyte count, total cholesterol, HDL cholesterol, ALT, and amylase. Blood gas analysis showed significant differences in pO_2_, SO_2_, and lactate between arterial and venous samples. This retrospective study provides statistical reference intervals for hematology, biochemistry, and blood gas analysis in clinically healthy long-tailed gorals, which is particularly valuable for endangered wildlife where obtaining an adequate number of specimens is challenging.

## Simple Summary

The long-tailed goral (*Naemorhedus caudatus*) is a small ungulate belonging to the subfamily Caprinae within the family Bovidae. Due to threats such as poaching, habitat loss, and extreme weather, its population has been gradually declining and is classified as an endangered species worldwide. In response, efforts are being made to conserve this endangered species through restoration programs, as well as rescuing gorals injured or stranded in the wild. In these conservation efforts, laboratory blood tests are very valuable for evaluating individual health status. The objective of this study is to establish species-specific reference intervals for blood test parameters of the long-tailed goral, contributing not only to clinical aspects but also to fundamental physiological research for restoration purposes.

## 1. Introduction

The long-tailed goral (*Naemorhedus caudatus*) is a small ungulate belonging to the order Artiodactyla, family Bovidae, and subfamily Caprinae within the genus *Naemorhedus* [1]. This species inhabits rocky, high-altitude mountainous regions in eastern Russia, northeastern China, and the Korean Peninsula [1]. In South Korea, they are mainly distributed in the steep mountain areas of Gangwon Province and northern Gyeongsangbuk Province [1]. Based on camera trapping and fecal analysis, an estimated 864–920 individuals inhabited South Korea in 2013 [2]. A 2022 report by the Korea National Park Institute confirmed 623 individuals within eight national parks [3]. Globally, the population is estimated at 2500–10,000 and is in decline. Consequently, the long-tailed goral is listed as a vulnerable species on the International Union for Conservation of Nature and Natural Resources (IUCN) Red List and is included in Appendix I of the Convention on International Trade in Endangered Species of Wild Flora and Fauna (CITES) as an internationally endangered species [1,4].

Major threats include habitat loss and fragmentation due to deforestation, road construction, climate change, and illegal poaching for traditional medicine and meat [4]. The species is especially vulnerable to heavy snowfall due to its short limbs and herbivorous diet, which limits foraging efficiency in deep snow [5]. For example, approximately 6000 individuals reportedly died between March 1964 and February 1965 following severe snowfall [6], and 24 individuals perished in Uljin County from March to June 2010 under similar conditions [7]. In response, the long-tailed goral has been protected as Natural Monument No. 217 since 1968 and designated as a first-class endangered species in South Korea since 1997. In 2006, the Korea National Park Institute for Wildlife Conservation (KNPI) established a restoration team that carries out habitat conservation, artificial reproduction, and the rescue, treatment, rehabilitation, and reintroduction of injured or weakened individuals.

The accurate diagnosis and health management of rescued or captive gorals require reference intervals for blood parameters derived from clinically healthy individuals. However, establishing such reference intervals is challenging due to the limited sample sizes typically available for endangered wildlife. Prior studies have been restricted by small sample sizes, limiting the statistical robustness of their findings [8,9,10]. Although reference intervals have been well established for other Caprinae species such as goats, sheep, and ibexes, substantial interspecies and interbreed variability limits their direct application to long-tailed gorals [11,12,13,14,15,16,17,18,19,20].

Therefore, the objective of this study was to establish comprehensive reference intervals for hematological, biochemical, and blood gas parameters in clinically healthy long-tailed gorals (*Naemorhedus caudatus*).

## 2. Materials and Methods

Blood test data were retrospectively reviewed from clinical records at the Northern Conservation Center of the Korea National Park Institute, located in Inje, Gangwon Province, South Korea. To ensure data accuracy, only blood samples from clinically healthy individuals were included based on a systematic review of veterinary records. A total of 75 long-tailed gorals met the inclusion criteria.

### 2.1. Animals

**Figure 1:**
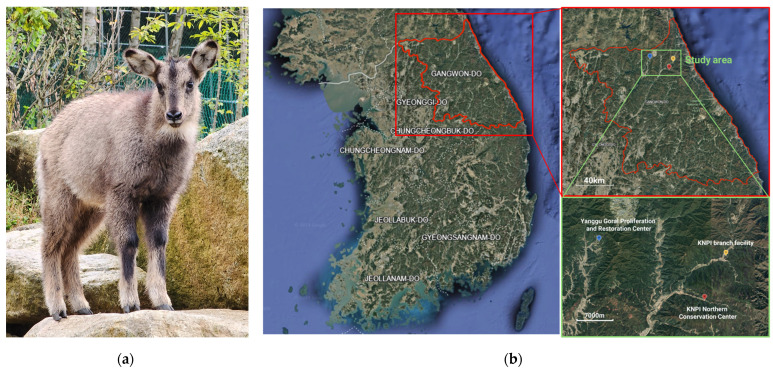
Subjects and locations of study: (**a**) long-tailed goral (*Naemorhedus caudatus*) in captivity; (**b**) locations of three facilities in Gangwon province.

The gorals were raised at three international endangered species breeding facilities in Gangwon Province, in the middle-east of the Korean peninsula, as part of endangered species restoration projects: the main facility of the Korea National Park Institute Northern Conservation Center (148 Han-gye-ri, Buk-myeon, Injegun, Gangwon-do, 38°07′25″ N, 128°18′24″ E, altitude 377 m), a branch facility of the Northern Conservation Center (San 35-3 Yongdae-ri, Buk-myeon, Inje-gun, Gangwon-do, 38°11′57″ N, 128°21′15″ E, altitude 438 m), and the Yanggu Goral Proliferation and Restoration Center (247 Pallang-ri, Dong-myeon, Yanggu-gun, Gangwon-do, 38°13′34″ N, 128°04′30″ E, altitude 433 m) (Figure 1). All gorals grazed in fenced outdoor enclosures on hillsides. Shelters were installed throughout the enclosures to protect the gorals from rain and sunlight. The primary feed provided was mulberry leaves, supplemented with fresh leaves from oak, acacia, and other seasonally available trees. Feed was provided once daily in the forenoon, and the gorals grazed on fresh grass growing in the enclosures. Fresh water was available ad libitum. The gorals’ weight ranged from 2.5 kg to 54.6 kg, and their ages from 0 to 15 years, including ten individuals with unknown ages. Ages were confirmed through medical records, and sex was determined during physical examination.

All individuals were anesthetized using a CO_2_-powered dart pistol with a barrel (PICO_2_ easy10-0, DANiNJECT, Kolding, Denmark) and anesthetic-loaded 5 mL dart syringe (s500, DANiNJECT, Denmark) in outdoor enclosures for annual routine health examinations or transmitter attachment prior to release. Three anesthesia protocols were used: medetomidine alone (0.07–0.1 mg/kg), a combination of medetomidine (0.07–0.1 mg/kg) and ketamine (2 mg/kg), and a combination of medetomidine (0.02 mg/kg) and Zoletil^®^ (Virbac SA, Carros, France) (2 mg/kg). Following anesthesia, all individuals were transported indoors for comprehensive health evaluations, including physical examination, body measurements, radiography, blood sampling, fecal examination, and screening for infectious diseases. Infectious disease tests included ELISA for foot-and-mouth disease and Johne’s disease, the Rose Bengal test for brucellosis, and rapid diagnostic kits for brucellosis, bovine tuberculosis, and five major pathogens associated with neonatal calf diarrhea (rotavirus, coronavirus, *Escherichia coli* K99, *Cryptosporidium*, and *Salmonella* spp.). All procedures, including animal capture, health assessments, and sample collection, were conducted by trained veterinarians and researchers. Blood test data from animals with abnormal clinical findings were excluded, and only one result per individual was included for each test type to avoid duplication. This study was conducted as a retrospective study using blood test data obtained during routine health screening procedures. Therefore, ethical approval was not required.

### 2.2. Sample Collection and Laboratory Analysis

Blood samples were collected from the jugular or cephalic vein and the femoral artery under anesthesia using 10–15 mL syringes with 18–20 gauge needles. Immediately after collection, blood was transferred into K_2_ ethylenediaminetetraacetic acid (EDTA) tubes and lithium heparin tubes, gently mixed by inversion, and stored at 4 °C. All blood analyses were performed within 12 h of sample collection.

For hematology analysis, anticoagulated venous blood from K_2_ EDTA tubes was utilized, and samples were analyzed using an automated veterinary hematology analyzer (MEK-6450K, Nihon Kohden, Tokyo, Japan). The following 20 parameters were determined: red blood cell count (RBC, 10^12^/L), hemoglobin (g/L), hematocrit (L/L), mean cell volume (MCV, fL), mean corpuscular hemoglobin (MCH, pg), mean corpuscular hemoglobin concentration (MCHC, g/L), red blood cell distribution width (RDW, %), white blood cell count (WBC, 10^9^/L), lymphocyte percent (%), monocyte percent (%), eosinophil percent (%), granulocyte percent (%), lymphocyte count (10^9^/L), monocyte count (10^9^/L), eosinophil count (10^9^/L), granulocyte count (10^9^/L), platelet count (10^9^/L), mean platelet volume (MPV, fL), and plateletcrit (L/L). The analysis procedures followed the instrument manual, and since species-specific settings for long-tailed gorals were not available, the test was conducted by setting the instrument for cows, taxonomically the most closely related species on the list.

For biochemistry testing, anticoagulated venous blood from lithium heparin tubes was used. Analyses were performed using a veterinary chemistry analyzer (DRI-CHEM 4000i, FUJIFILM, Tokyo, Japan). The following 27 parameters were determined: total protein (g/L), albumin (g/L), glucose (mmol/L), triglyceride (mmol/L), total cholesterol (T-Chol, mmol/L), high-density lipoprotein cholesterol (HDL-Chol, mmol/L), aspartate aminotransferase (AST, U/L), alanine aminotransferase (ALT, U/L), alkaline phosphatase (ALP, U/L), gamma-glutamyltransferase (GGT, U/L), lactate dehydrogenase (LDH, U/L), creatine phosphokinase (CPK, U/L), creatine kinase MB isoenzyme (CK-MB, U/L), amylase (U/L), lipase (U/L), total bilirubin (T-Bil, μmol/L), direct bilirubin (D-Bil, μmol/L), blood urea nitrogen (BUN, mmol/L), creatinine (μmol/L), uric acid (µmol/L), ammonia (NH_3_, μmol/L), calcium (Ca, mmol/L), inorganic phosphorus (IP, mmol/L), magnesium (Mg, mmol/L), sodium (Na, mmol/L), potassium (K, mmol/L), and chloride (Cl, mmol/L).

For blood gas analysis, whole blood was drawn directly from the syringe into the cartridge of the epoc Blood Analysis System (Siemens Healthineers, Forchheim, Germany) and blood was immediately injected into the testing kit from the syringe containing whole blood, following the manufacturer’s protocol. The following parameters were determined: pH, partial pressure of carbon dioxide (pCO_2_, kPa), partial pressure of oxygen (pO_2_, kPa), bicarbonate (HCO_3_, mmol/L), base excess in extracellular fluid (BEecf, mmol/L), saturated oxygen (SO_2_, %), total oxygen content (TO_2_, mmol/L), anion gap (AnGap), and lactate (mmol/L).

### 2.3. Statistical Analysis

All statistical procedures were performed in accordance with the guidelines for establishing reference intervals published by the American Society for Veterinary Clinical Pathology (ASVCP) [21]. Blood test data were extracted from electronic medical records and organized using Microsoft Excel.

Outlier detection and reference interval calculation were conducted using the Reference Value Advisor V2.1 program [22]. Outliers were identified based on visual inspection of histograms and Q–Q plots, and further evaluated using Dixon’s range test and Horn’s algorithm based on Tukey’s interquartile fences. Outliers were excluded for each parameter, and the distribution of the remaining data was re-evaluated. Reference intervals were defined as the central 95% of values from a healthy reference population [21]. Distributional characteristics of the data were assessed using the Anderson–Darling test and a robust test for normality. Most data were transformed using the Box–Cox method to approximate a normal distribution prior to reference interval calculation. A parametric bootstrap method was primarily applied to compute the reference intervals. If the Box–Cox transformation did not result in a distribution approximating normality, or if it worsened the distributional characteristics, the parametric bootstrap method was applied without transformation. For datasets with ≥40 samples that failed to meet both normality and symmetry assumptions, the non-parametric bootstrap method was applied to estimate the 2.5th to 97.5th percentiles.

The influence of age and sex was assessed for hematology and biochemistry parameters, while differences between arterial and venous blood were examined for blood gas analysis. Statistical analysis using the SPSS program, version 27, was conducted to determine significant differences between each group. Between-group mean comparisons were validated using the Mann–Whitney U test, nonparametric method, due to the small size of each group. Results with *p* < 0.05 were considered statistically significant.

## 3. Results

### 3.1. Descriptive Analysis of the Total Number of Animals

Blood test results from 75 long-tailed gorals (41 males, 34 females) were analyzed, including 68 hematology profiles, 75 biochemistry profiles, 32 arterial blood gas analyses, and 32 venous blood gas analyses. Gorals aged three years or older were classified as adults (51/75), and those younger than three years were classified as young (17/75). The age of seven individuals was unknown; they were included in the calculation of reference intervals but excluded from group comparisons.

### 3.2. Reference Intervals

Hematological reference intervals for long-tailed gorals are presented in Table 1 (*n* = 57 to 68).

For biochemistry variables, the results are presented in Table 2 (*n* = 13 to 75).

For arterial and venous blood gas analysis, the results are presented in Table 3 and Table 4 (*n* = 29 to 32).

### 3.3. Influence of Age and Sex

The influence of age and sex on hematology and biochemistry results is presented in Table 5 and Table 6. The influence of sex was analyzed only in sexually mature gorals, older than three years.

The values of MCV, MCH, amylase, and creatinine were significantly (*p* < 0.05) higher in adults, while platelet, plateletcrit, lymphocyte count, ALP, LDH, Ca, IP, T-Chol and HDL-Chol values were significantly (*p* < 0.05) higher in the young group.

The values of RBC, ALT, GGT, amylase were significantly (*p* < 0.05) higher in male gorals, and the values of WBC, lymphocyte count, T-Chol, HDL-Chol were significantly (*p* < 0.05) higher in female gorals.

### 3.4. Comparison of Arterial and Venous Blood Gas Analysis Results

Among blood gas parameters, pO_2_ and SO_2_ were significantly higher in arterial blood, while lactate was significantly higher in venous blood, as shown in Table 7.

## 4. Discussion

### 4.1. Reference Intervals for Hematology, Biochemistry Parameters, and Blood Gas Analysis

The hematological reference intervals established in this study were broadly consistent with those previously reported in domestic goats and wild Caprinae species [8,9,10,11,12,13,14,15,16,17,18]. Leukocyte and differential counts exhibited wider distributions than earlier goral studies, likely due to the larger and more diverse sample set in the present research [8,9,10,11]. MCV values were similar to those in goats and sheep, which have the smallest erythrocytes among domestic animals [23]. As impedance-based hematology analyzers may misclassify small erythrocytes, the interpretation of red cell indices in gorals requires caution [23].

Biochemical profiles also generally aligned with those reported for other Caprinae species [8,9,10,12,13,14,15,16,17,18]. Lipid values, including triglycerides and cholesterol fractions, were lower but had wide reference intervals, possibly reflecting variability in nutrition, season, stress, or hormonal status [13,14,15,24,25,26]. Among liver enzymes, AST and ALT were lower than in previous goral studies [8,9], although ALT is known to be nonspecific in ruminants [27]. In contrast, ALP activity was elevated, likely due to the inclusion of younger animals [28]. Amylase activity was higher than previously reported and may be influenced by dietary composition [8,9,29,30]. These enzymes had considerable inter-individual variation. Outlier exclusion improved reference interval precision. Enzyme values outside these intervals do not necessarily indicate disease and must be interpreted within their clinical context [31]. Other biochemical values were comparable to prior reports [29,30].

This study is the first to report reference intervals for several analytes in long-tailed gorals, including HDL-cholesterol, LDH, CPK, CK-MB, lipase, D-bilirubin, uric acid, ammonia, and magnesium. Among these, LDH, CPK, uric acid, and magnesium were within the expected ranges reported for other Caprinae [12,13,14,15,16,17,18,32,33]. Although data on CK-MB in small ruminants are limited, the observed activities resembled baseline values in goat cardiac biomarker studies, suggesting potential utility in clinical monitoring [34,35,36]. Blood ammonia (NH_3_) concentrations were lower than in hyperammonemia control groups in goats, though no reference ranges are currently established for small ruminants [37]. Further research is warranted to validate the diagnostic value of these parameters in long-tailed gorals.

Reference intervals for blood gas parameters were also reported for the first time. Compared to domestic goats (*Capra hircus*) and Arabian oryx (*Oryx leucoryx*), gorals showed higher pCO_2_ and lower pO_2_ values, while pH, HCO_3_^−^, and BEecf were within similar ranges [18,19,20]. These differences may reflect species-specific respiratory physiology or variations in sampling conditions. As samples were obtained under field anesthesia without standardized oxygen supplementation or controlled timing, future studies are needed to refine these parameters under standardized protocols.

### 4.2. Influence of Age and Sex on Hematology and Biochemistry Results

Young gorals had higher ALP activity, calcium, and inorganic phosphorus concentrations, consistent with active bone growth and increased metabolic demands during development [38,39,40]. Total and HDL-cholesterol concentrations were higher in adult females, potentially due to estrogen-mediated lipid regulation [41]. These lipid parameters also appeared higher in young individuals; however, sex distribution differences—more females in the young group and more males in the adult group—suggest that the observed age effects may reflect sex-related differences rather than age alone.

Amylase activity was higher in adults, particularly in males, whereas ALT and GGT activities did not differ by age but were higher in males. Similar trends in amylase and ALT have been reported in Persian wild goats (*Capra aegagrus*) and Hair goats (*Capra hircus*) [12,16]. However, given the high inter-individual variability in these enzyme activities, further research with larger and stratified sample sizes will be needed to better clarify the effects of age and sex.

### 4.3. Evaluation of Arterial and Venous Blood Gas Differences

As expected, arterial pO_2_ and SO_2_ values were markedly higher than venous values, confirming that venous samples are inappropriate for evaluating these analytes [42]. Venous lactate concentrations were significantly higher, likely due to transient hypoxia or exertion during capture and transport under anesthesia without immediate oxygen supplementation [43]. These factors may have contributed to the observed arterial–venous lactate gradient.

### 4.4. Potential Effects of Captivity, Capture Method, and Anesthetic Protocol

The interpretation of hematologic and biochemical data in wildlife must consider environmental and procedural variables. Although the gorals in this study were housed in semi-natural outdoor enclosures, captivity can still induce physiological changes not observed in free-ranging animals. Captive individuals may experience chronic stress and heightened responsiveness to stimuli, as indicated by elevated RBC counts, hemoglobin concentrations, and hematocrit values likely associated with splenic contraction triggered by catecholamine release, as reported in captive Iberian ibex (*Capra pyrenaica*) and roe deer (*Capreolus capreolus*) [44,45]. Additionally, reduced physical activity in captive animals has been linked to sharper increases in muscle-related enzymes during capture compared to wild individuals [45].

Seasonal variation is another key factor. Shifts in energy balance and metabolic activity across seasons have been shown to influence blood values in wild ruminants, including the Iberian ibex and southern chamois (*Rupicapra pyrenaica*) [14,46,47,48]. Although most samples in this study were collected in late autumn (non-breeding season), sampling was not fully standardized, and seasonal effects cannot be ruled out.

Stress related to capture and handling may also impact results. Activation of the hypothalamic–pituitary–adrenal (HPA) axis during physical restraint can elevate glucose, lactate, and muscle enzymes (CK, LDH, AST), as well as altering leukocyte profiles [49,50,51,52]. Efforts were made to minimize pursuit time before darting to reduce stress, yet variability in lactate concentrations and enzyme activities may still reflect individual behavioral responses.

Additionally, anesthesia can influence blood parameters. Prior studies in ruminants have shown increased lactate and metabolic acidosis, as well as reduced urea and creatinine under anesthesia [53]. While xylazine–ketamine and medetomidine–ketamine appear comparable in Iberian ibex [54], the protocols used in this study—medetomidine alone, medetomidine–ketamine, and medetomidine–zolazepam–tiletamine—differ in pharmacologic profiles. Although limited sample size precluded statistical comparison, the potential impact of anesthetic protocol on clinical values warrants further investigation.

To improve the robustness and clinical utility of reference intervals, future research should incorporate standardized sampling conditions and controlled comparisons across anesthetic regimens.

## 5. Conclusions

This study determined comprehensive reference intervals for hematology, biochemistry, and blood gas parameters in clinically healthy long-tailed gorals, marking a significant contribution to the limited data available for this endangered species. The large sample size also enabled analyses of differences based on sex and age. Reference intervals for blood gas parameters are presented for the first time in long-tailed gorals, along with several previously unreported biochemical markers. The findings can contribute to improved clinical management for this species and serve as a foundation for future studies in this and other endangered species.

## Figures and Tables

**Table 1:** Reference intervals for hematology values of long-tailed goral.

Parameters	*n*	Mean ± SD	Median	Min	Max	Reference Interval	Lower Limit (90% CI)	Upper Limit (90% CI)
RBC (10^12^/L)	68	11.41 ± 1.59	11.55	7.64	14.50	8.21–14.60	7.67–8.69	14.01–15.10
Hemoglobin (g/L)	68	105 ± 16	105	69	139	74–139	68–79	132–145
Hematocrit (L/L)	68	0.34 ± 0.05	0.34	0.25	0.44	0.25–0.45	0.23–0.27	0.43–0.46
MCV (fL)	68	30.4 ± 3.3	30.3	25.0	39.2	24.8–38.2	24.1–25.5	36.5–40.0
MCH (pg)	68	9.3 ± 1.0	9.2	7.5	11.6	7.2–11.4	6.9–7.5	11.0–11.8
MCHC (g/L)	61	307 ± 8	307	284	323	289–322	286–292	319–324
RDW (%)	63	17.1 ± 0.9	17.0	15.1	19.3	15.5–19.1	15.3–15.8	18.7–19.6
WBC (10^9^/L)	66	6.1 ± 2.3	5.6	2.9	12.4	2.9–11.8	2.6–3.2	10.5–13.4
Lymphocyte (%)	67	38.6 ± 11.5	38.6	15.7	66.7	15.4–61.9	11.6–19.4	57.4–66.0
Monocyte (%)	62	0.5 ± 0.7	0.2	0	3.0	0–2.8	0–0	1.9–3.0
Eosinophil (%)	65	6.3 ± 4.4	6.1	0	15.3	0–14.3	0–0	13.4–15.3
Granulocyte (%)	66	53.8 ± 13.3	53.2	20.7	84.3	27.0–80.6	22.4–31.4	75.7–85.3
Lymphocyte (10^9^/L)	67	2.3 ± 0.8	2.3	1.1	4.2	1.0–4.0	0.8–1.1	3.7–4.3
Monocyte (10^9^/L)	64	0.03 ± 0.05	0	0	0.2	0–0.2	0–0	0.1–0.2
Eosinophil (10^9^/L)	66	0.4 ± 0.3	0.4	0	1.1	0–1.0	0–0	0.8–1.1
Granulocyte (10^9^/L)	64	3.3 ± 1.8	2.8	1.1	8.0	1.2–8.9	1.0–1.3	7.1–11.2
Platelet (10^9^/L)	65	355 ± 116	340	159	666	180–627	167–198	562–686
Plateletcrit (L/L)	64	0.0017 ± 0.0010	0.0015	0.0006	0.0042	0.0006–0.0040	0.0006–0.0006	0.0034–0.0042
MPV (fL)	65	4.4 ± 1.5	4.3	2.3	7.8	2.4–7.7	2.3–2.6	7.4–7.8
PDW (%)	57	16.1 ± 1.0	16.2	13.6	18.0	13.8–17.9	13.3–14.4	17.6–18.2

SD, standard deviation; Min, minimum; Max, maximum; CI, confidence interval; RBC, red blood cell; MCV, mean cell volume; MCH, mean corpuscular hemoglobin; MCHC, mean corpuscular hemoglobin concentration; RDW, red blood cell distribution width; WBC, white blood cell; MPV, mean platelet volume; PDW, platelet distribution width.

**Table 2:** Reference intervals for biochemistry and electrolyte values of long-tailed goral.

Parameters	*n*	Mean ± SD	Median	Min	Max	Reference Interval	Lower Limit (90% CI)	Upper Limit (90% CI)
Total protein (g/L)	73	59 ± 5	59	48	70	49–68	48–51	67–70
Albumin (g/L)	71	35 ± 3	35	26	41	27–41	26–29	40–41
Glucose (mmol/L)	74	7.94 ± 2.89	8.05	2.77	14.21	2.44–13.98	1.66–3.27	12.93–15.04
Triglyceride (mmol/L)	69	0.05 ± 0.06	0.01	0.01	0.25	0.01–0.24	0.01–0.01	0.16–0.25
T-Chol (mmol/L)	72	0.96 ± 0.39	0.88	0.31	1.91	0.34–1.91	0.28–0.41	1.71–2.12
HDL-Chol (mmol/L)	70	0.83 ± 0.36	0.80	0.23	1.60	0.26–1.63	0.21–0.34	1.45–1.78
AST (U/L)	68	127 ± 41	117	62	247	69–236	64–76	206–269
ALT (U/L)	72	59 ± 15	57	30	100	34–94	31–37	87–101
ALP (U/L)	64	469 ± 170	414	238	904	235–904	214–259	786–1038
GGT (U/L)	72	216 ± 66	205	92	366	96–356	82–112	330–382
LDH (U/L)	66	549 ± 136	542	295	840	276–824	223–331	779–872
CPK (U/L)	55	338 ± 173	319	135	852	117–767	105–139	639–890
CK-MB (U/L)	55	153 ± 58	144	47	292	37–272	12–60	248–293
Amylase (U/L)	71	152 ± 62	146	30	289	41–286	25–56	261–313
Lipase (U/L)	13	49 ± 8	50	39	62	32–68	26–68	60–76
T-bil (μmol/L)	69	8.55 ± 8.55	6.84	1.71	32.49	1.71–32.49	1.71–3.42	27.36–32.49
D-bil (μmol/L)	69	3.42 ± 3.42	1.71	1.71	13.68	1.71–11.97	1.71–1.71	11.97–13.68
BUN (mmol/L)	73	8.85 ± 3.11	8.57	2.25	16.53	2.64–15.07	1.71–3.61	14.03–16.03
Creatinine (μmol/L)	69	114.9 ± 26.5	106.1	53.0	176.8	53.0–168.0	44.2–61.9	159.1–185.6
Uric acid (μmol/L)	71	17.84 ± 5.95	17.84	11.90	23.79	11.90–23.79	11.90–11.90	23.79–23.79
NH_3_ (μmol/L)	63	5.90 ± 3.28	4.70	1.88	15.41	1.71–14.52	1.53–2.06	12.11–16.58
Ca (mmol/L)	74	2.27 ± 0.23	2.25	1.68	2.70	1.80–2.73	1.73–1.90	2.65–2.80
IP (mmol/L)	69	1.42 ± 0.61	1.35	0.42	3.16	0.45–2.84	0.39–0.58	2.52–3.19
Mg (mmol/L)	72	1.19 ± 0.21	1.19	0.70	1.81	0.86–1.65	0.78–1.19	1.56–1.77
Na (mmol/L)	75	141 ± 6	141	127	155	131–153	129–132	151–156
K (mmol/L)	74	4.8 ± 0.8	4.8	3.1	6.7	3.2–6.4	2.9–3.4	6.2–6.7
Cl (mmol/L)	75	100 ± 7	99	80	116	86–115	84–89	112–118

SD, standard deviation; Min, minimum; Max, maximum; CI, confidence interval; T-Chol, total cholesterol; HDL-Chol, high-density lipoprotein cholesterol; AST, aspartate aminotransferase; ALT, alanine aminotransferase; ALP, alkaline phosphatase; GGT, gamma-glutamyltransferase; LDH, lactate dehydrogenase; CPK, creatine phosphokinase; CK-MB, creatine kinease MB isoenzyme; T-bil, total bilirubin; D-bil, direct bilirubin; BUN, blood urea nitrogen; IP, inorganic phosphorus.

**Table 3:** Reference intervals for arterial blood gas parameters and lactate of long-tailed goral.

Parameters	*n*	Mean ± SD	Median	Min	Max	Reference Interval	Lower Limit (90% CI)	Upper Limit (90% CI)
pH	32	7.35 ± 0.05	7.36	7.24	7.45	7.24–7.46	7.21–7.26	7.43–7.49
pCO_2_ (kPa)	32	7.13 ± 1.07	6.93	5.32	9.97	5.36–9.81	5.07–5.72	8.96–10.74
pO_2_ (kPa)	30	9.30 ± 2.08	8.73	6.97	15.68	6.93–15.66	6.77–7.18	12.96–19.00
HCO_3_ (mmol/L)	32	28.8 ± 2.8	28.7	25.1	35.5	24.9–35.5	24.2–25.7	33.0–38.6
BEecf (mmol/L)	32	3.5 ± 2.7	3.4	−2.1	10.5	−1.7–9.3	−2.7–−0.4	7.6–10.7
SO_2_ (%)	32	89.9 ± 5.5	90.4	81.7	99.4	78.5–100.0	75.4–81.2	98.7–104.2
TCO_2_ (mmol/L)	31	30.1 ± 2.3	29.9	26.8	35.6	27.0–38.8	26.8–27.2	35.6–43.2
AnGap	29	8 ± 4	9	−2	16	0–17	−2–2	14–19
Lactate (mmol/L)	29	0.65 ± 0.28	0.60	0.30	1.24	0.25–1.49	0.20–0.31	1.16–1.93

SD, standard deviation; Min, minimum; Max, maximum; CI, confidence interval; pCO_2_, partial pressures of carbon dioxide; pO_2_, partial pressures of oxygen; BEecf, base excess in extracellular fluid; SO_2_, saturation of hemoglobin with oxygen; TCO_2_, total carbon dioxide; AnGap, anion gap.

**Table 4:** Reference intervals for venous blood gas parameters and lactate of long-tailed goral.

Parameters	*n*	Mean ± SD	Median	Min	Max	Reference Interval	Lower Limit (90% CI)	Upper Limit (90% CI)
pH	32	7.34 ± 0.06	7.34	7.21	7.47	7.22–7.47	7.19–7.25	7.44–7.51
pCO_2_ (kPa)	32	6.85 ± 0.97	6.86	4.76	8.92	4.93–8.96	4.48–5.43	8.44–9.54
pO_2_ (kPa)	32	5.37 ± 1.17	5.01	3.45	8.10	4.80–8.37	3.20–3.79	7.36–9.45
cHCO_3_ (mmol/L)	32	27.7 ± 3.8	27.8	20.6	36.8	20.2–35.9	18.4–22.0	33.7–37.9
BEecf (mmol/L)	32	2.1 ± 4.4	2.6	−6.4	11.9	−6.9–11.1	−9.2–−4.6	8.9–13.5
SO_2_ (%)	32	67.7 ± 12.4	65.9	45.0	90.6	42.1–93.4	36.5–48.8	87.7–99.1
TCO_2_ (mmol/L)	32	28.9 ± 3.5	29.1	22.0	35.7	21.7–36.2	20.0–23.6	34.3–37.9
AnGap	32	10 ± 6	11	−3	17	−2–21	−5–1	18–24
Lactate (mmol/L)	31	2.50 ± 1.68	2.16	0.47	5.94	0–7.03	−0.33–0.40	5.39–8.90

SD, standard deviation; Min, minimum; Max, maximum; CI, confidence interval; pCO_2_, partial pressures of carbon dioxide; pO_2_, partial pressures of oxygen; BEecf, base excess in extracellular fluid; SO_2_, saturation of hemoglobin with oxygen; TCO_2_, total carbon dioxide; AnGap, anion gap.

**Table 5:** Hematological and biochemical parameters significantly affected by age in long-tailed goral.

Parameters	Young (<3 y)			Adult (≥3 y)			Statistical Significance *
	*n*	Mean ± SD	RI	*n*	Mean ± SD	RI	
MCV (fL)	17	29.0 ± 3.6	19.1–35.8	42	31.4 ± 3.0	24.7–37.2	++
MCH (pg)	17	8.7 ± 1.1	7.4–13.4	42	9.6 ± 1.0	7.2–11.2	++
Lymphocyte (10^9^/L)	17	2.7 ± 0.9	0.9–4.7	41	2.0 ± 0.6	0.9–3.3	++
Platelet (10^9^/L)	14	400 ± 127	165–720	42	317 ± 96	182–578	+
Plateletcrit (L/L)	13	0.0018 ± 0.0008	0.0005–0.0042	42	0.0014 ± 0.0008	0.0005–0.0049	+
T-Chol (mmol/L)	14	1.24 ± 0.36	0.47–2.04	48	0.88 ± 0.39	0.31–1.89	++
HDL-Chol (mmol/L)	14	1.09 ± 0.31	0.59–2.09	46	0.75 ± 0.36	0.23–1.68	++
ALP (U/L)	8	762 ± 152	403–1283	46	418 ± 122	222–704	++
LDH (U/L)	12	613 ± 139	295–931	46	529 ± 125	313–818	+
Amylase (U/L)	14	115 ± 33	41–190	48	165 ± 64	35–295	++
Creatinine (μmol/L)	13	88.4 ± 26.5	44.2–168.0	49	114.9 ± 26.5	53.0–176.8	++
Ca (mmol/L)	13	2.42 ± 0.17	2.10–2.90	51	2.23 ± 0.23	1.77–2.65	++
IP (mmol/L)	14	1.94 ± 0.48	1.00–3.23	46	1.26 ± 0.58	0.45–2.90	++

SD, standard deviation; RI, reference interval; MCV, mean cell volume; MCH, mean corpuscular hemoglobin; T-Chol, total cholesterol; HDL-Chol, high-density lipoprotein cholesterol; ALP, alkaline phosphatase; LDH, lactate dehydrogenase; IP, inorganic phosphorus. * Statistical significance indicates: +, moderate significance (0.01 < *p* < 0.05); ++, strong significance (*p* < 0.01).

**Table 6:** Hematological and biochemical parameters significantly affected by sex in adult long-tailed goral.

Parameters	Adult Male			Adult Female			Statistical Significance **
	*n*	Mean ± SD	RI	*n*	Mean ± SD	RI	
RBC (10^12^/L)	27	11.57 ± 1.73	6.89–14.52	15	10.55 ± 1.64	7.17–14.41	+
WBC (10^9^/L)	26	5.3 ± 2.3	2.5–11.8	15	6.5 ± 2.6	*	+
Lymphocyte (10^9^/L)	26	1.8 ± 0.5	0.9–3.0	15	2.4 ± 0.6	1.1–3.6	++
T-Chol (mmol/L)	31	0.67 ± 0.21	0.28–1.16	17	1.22 ± 0.39	0.39–2.07	++
HDL-Chol (mmol/L)	29	0.59 ± 0.23	0.21–1.11	17	1.06 ± 0.34	0.31–1.81	++
ALT (U/L)	32	62 ± 13	41–95	17	49 ± 10	28–72	++
GGT (U/L)	32	233 ± 62	126–379	18	189 ± 61	71–337	+
Amylase (U/L)	31	187 ± 59	64–309	17	126 ± 54	16–251	++

SD, standard deviation; RI, reference interval; RBC, red blood cell; WBC, white blood cell; T-Chol, total cholesterol; HDL-Chol, high-density lipoprotein cholesterol; ALT, alanine aminotransferase; GGT, gamma-glutamyltransferase. * Non-computable. ** Statistical significance indicates: +, moderate significance (0.01 < *p* < 0.05); ++, strong significance (*p* < 0.01).

**Table 7:** Statistically significant differences between the mean arterial and venous blood gas parameters in long-tailed goral.

Parameters	Arterial Blood		Venous Blood		Statistical Significance *
	*n*	Mean ± SD	*n*	Mean ± SD	
pO_2_ (kPa)	30	9.30 ± 2.08	32	5.37 ± 1.17	++
SO_2_ (%)	32	89.9 ± 5.5	32	67.7 ± 12.4	++
Lactate (mmol/L)	29	0.65 ± 0.28	31	2.50 ± 1.68	++

SD, standard deviation; pO_2_, partial pressures of oxygen; SO_2_, saturation of hemoglobin with oxygen. * Statistical significance indicate: ++, strong significance (*p* < 0.01).

## Data Availability

The data that support the findings of this retrospective study are available upon reasonable request to the authors.
